# Editorial: The Claustrum: charting a way forward for the brain's most mysterious nucleus

**DOI:** 10.3389/fnsys.2015.00103

**Published:** 2015-07-15

**Authors:** Ariel Y. Deutch, Brian N. Mathur

**Affiliations:** ^1^Departments of Psychiatry and Pharmacology, Vanderbilt University Medical CenterNashville, TN, USA; ^2^Department of Pharmacology, University of Maryland School of MedicineBaltimore, MD, USA

**Keywords:** insula, insular cortex, prefrontal cortex, cingulate cortex, attention, conciousness, comparative anatomy, connectivity

There are many reasons why investigators decide to expend their energy on studying a particular nucleus in the brain. Sometimes the choice of structures has little to do with new insights that may be gleaned but is more attributable to the tractability of the site to experimental manipulation. However, some sites remain relatively obscure because their structure almost seems designed to impede research. The claustrum belongs to the latter group. Entombed between tightly constricting white matter bundles, the claustrum both escaped and resisted significant attention for much of the twentieth century. However, over the past decade there has been a resurgence in interest in the claustrum. This renewed attention is in large part attributable to a paper by Crick and Koch (2005), which advanced the hypothesis that the claustrum plays a central role in binding and consciousness. Seductively written, and with Crick and Koch's imprimatur, a flurry of studies ensued. While it appeared that this opened the door to experimental studies of claustral function, over the past decade it has become apparent that the door is merely ajar.

This collection of papers is an attempt to bring together many of the key recent findings on the claustrum. Perusing the volume quickly reveals that there is a striking emphasis on the anatomy of the claustrum, with most contributions emphasizing comparative anatomy of the claustrum, localization of different types of chemically-coded neurons within the claustrum, and new details on the morphology of claustral neurons. This selective attention to the anatomy of the claustrum has led some wags to comment that the anatomy of the claustrum should by now have been defined. However, striking differences in claustral organization and the anatomy of the region surrounding the claustrum across various species led to confusion regarding the boundaries of the claustrum. This resulted in some definitions being overly inclusive, adding to the claustrum several cortical regions that are now considered to be distinct. Other definitions were too restrictive and did not account for the ontogeny of the claustrum. This situation led Crick and Koch (2005) to decry the lack of objective criteria for the definition of the claustrum. The past decade has mainly been spent trying to respond to their plea.

This collection of papers appears to have arrived at a consensus regarding the definition of the claustrum, as seen from the vantage point of anatomical studies. Although there remain some small bones of contention, these are relatively minor and will no doubt soon be resolved. In light of the evolutionary expansion of the claustrum to its (discontinuous) presence in humans and other animals with large cortices, it is not surprising that about half of the manuscripts involve studies of primates (Baizer et al., 2014; Gattass et al., 2014; Hinova-Palova et al., 2014; Johnson et al., 2014; Pirone et al., 2014; Reser et al., 2014), with two papers on cetaceans (Baizer et al., 2014; Cozzi et al., 2014), and still others on rodents (Rockland, 2014; Patzke et al., 2014; Watakabe et al., 2014). There are somewhat fewer papers on the transmitters and molecules present in different populations of claustral cells (Cozzi et al., 2014; Hinova-Palova et al., 2014; Pirone et al., 2014; Rockland, 2014; Watakabe et al., 2014).

Baizer and colleagues, in their paper on the structure of the claustrum in higher order species, reminds us to ask “what does structure tell us about function?,” a query echoed by Mathur (2014) in his review. In addition to the aesthetic qualities of neuroanatomical preparations, neuroscience labors under the assumption that structure defines function, and that understanding morphology and hodology allows us to formulate testable hypotheses of neural function. In the current collection of papers, intended as a survey of the claustrum, only the Smith and Alloway (2014) contribution and that of Remedios et al. (2014) empirically explore the functions of the claustrum. We are obviously at a crossroads in claustral research, where our knowledge of the anatomy is sufficient to warrant testing the hypotheses of claustral function that almost every paper in this collection have advanced.

As noted earlier, the claustrum's peculiar shape has historically been a major impediment to functional studies of the claustrum. Fortunately, the advent of new methods and improvements in existing methods has made it possible to probe claustral function at the molecular, physiological, and behavioral levels. One can now study the physiology of the claustrum using optogenetic or chemogenetic approaches in slice preparations and *in vivo*, and image the activity of subpopulations of genetically defined claustral neurons using calcium imaging in awake, behaving animals. The use of laser capture microdissection and other methods such as immunopanning permits one to obtain claustral neurons without fear of contamination from adjacent structures, and analyze the proteome or transcriptome of these cells; this can even be done in claustral cells identified as projecting to specific targets. Non-invasive *in vivo* imaging methods can now be applied to both human and non-human studies: the resolution of structural imaging is now sufficient (7T) to undertake longitudinal studies of claustrum volume in health and disease, and with co-registration algorithms it may be possible to differentiate the contribution of the claustrum and insular cortex to fMRI signal changes. Finally, advances in methods for producing knockout and transgenic animals, such as that afforded by CRISPR/cas approaches, has made the rapid generation of such animals possible, opening the door to behavioral studies of claustral function.

Over the decade since the publication of Crick and Koch's cry for molecular studies of the claustrum and their hypothesis on claustral involvement in binding of percepts, a consensus regarding claustral structure has been reached. Over the next decade we look forward to a comparable blossoming of functional studies that unlock the deep secrets of the claustrum.

## Conflict of interest statement

The authors declare that the research was conducted in the absence of any commercial or financial relationships that could be construed as a potential conflict of interest.
